# Association Between Visceral Fat and Lung Function Impairment in Overweight and Grade I Obese Women: A Cross-Sectional Study

**DOI:** 10.3390/arm92060048

**Published:** 2024-12-18

**Authors:** Anamei Silva-Reis, Boris Brill, Maysa Alves Rodrigues Brandao-Rangel, Renilson Moraes-Ferreira, Dobroslav Melamed, Helida Cristina Aquino-Santos, Claudio Ricardo Frison, Regiane Albertini, Rodrigo Álvaro Brandao Lopes-Martins, Luís Vicente Franco de Oliveira, Gustavo Paixao-Santos, Carlos Rocha Oliveira, Asghar Abbasi, Rodolfo P. Vieira

**Affiliations:** 1 Laboratory of Pulmonary and Exercise Immunology (LABPEI), Evangelical University of Goiás (UniEvangélica), Avenida Universitária Km 3,5, Anápolis 75083-515, GO, Brazil; anameisreis97@gmail.com (A.S.-R.); ralopesmartins@gmail.com (R.Á.B.L.-M.); oliveira.lvf@gmail.com (L.V.F.d.O.); vibesgustavo@gmail.com (G.P.-S.); 2 Post-Graduation Program in Sciences of Human Movement and Rehabilitation, Federal University of Sao Paulo, Avenida Ana Costa 95, Santos 11060-001, SP, Brazil; maysarangel_4@hotmail.com (M.A.R.B.-R.); renilsonmoraesferreira@gmail.com (R.M.-F.); helidacristina.fisio@yahoo.com.br (H.C.A.-S.); claudiofrison@gmail.com (C.R.F.); regiane.albertini@unifesp.br (R.A.); 3 Leniado Medical Center, Divrei Khayim St. 16, Nethanya 4244916, Israel; drborisbrill@gmail.com; 4 Department of Research and Development, Libi Pharm, Ben Gurion 70, Rechovot 7639461, Israel; dobroslav.melamed@gmail.com; 5 GAP Biotech Laboratory of Biotechnology and Bioinformatics, Rua Comendador Remo Cesaroni 223, São José dos Campos 12243-020, SP, Brazil; pharmacologia@hotmail.com; 6 Division of Respiratory and Critical Care Physiology and Medicine, Department of Medicine, The Lundquist Institute for Biomedical Innovation at Harbor-UCLA Medical Center, 1124 W Carson St, CDCRC Building, Torrance, CA 90502, USA; asghar.abbasi@lundqusit.org; 7 Brazilian Institute of Teaching and Research in Pulmonary and Exercise Immunology (IBEPIPE), Rua Pedro Ernesto 240, São José dos Campos 12245-520, SP, Brazil

**Keywords:** overweight, obesity, visceral fat, lung function, inflammation, lung mechanics

## Abstract

**Highlights:**

**What are the main findings?**

- Visceral fat is more related to impairment of the lung function and mechanics than subcutaneous or total fat in women.
- Visceral fat strong correlates to impairment of pulmonary immune response in overweight and obese women.
- Visceral fat strong correlates to impairment of systemic immune response in overweight and obese women.

**Abstract:**

Beyond the common comorbidities related to obesity, such as type 2 diabetes and cardiovascular diseases, impaired lung function is already known, but whether the fat distribution (sub-cutaneous, visceral) affects the lung function and pulmonary immune response are poorly known. Few evidence has shown that visceral fat is associated with insulin resistance, low-grade inflammation, and reduced lung function. In the present study, the body composition and fat distribution were evaluated by multi-frequency octopolar bioimpedance. This study demonstrated a possible association of increased visceral fat with impaired lung function in obesity grade I (n = 28; 45.46 ± 10.38 years old) women that was not observed in normal weight (n = 20; 43.20 ± 10.78 years old) and in overweight women (n = 30; 47.27 ± 10.25 years old). We also identified a negative correlation in FVC% (R^2^ = 0.9129; *p* < 0.0236), FEV1% (R^2^ = 0.1079; *p* < 0.0134), PEF% (R^2^ = 0.1673; *p* < 0.0018), and VC IN% (R^2^ = 0.1330; *p* < 0.0057) in the obesity grade I group, clearly demonstrating that higher levels of visceral fat correlate with reduced lung function, but not with sub-cutaneous fat. In addition, for the first time, a negative correlation among anti-fibrotic protein klotho (R^2^ = 0.09298; *p* < 0.0897) and anti-inflammatory IL-10 (R^2^ = 0.1653; *p* < 0.0487) in plasma was observed, in contrast to increased visceral fat. On the contrary, in breath condensate, a positive correlation for adiponectin (R^2^ = 0.5665; *p* < 0.0120), IL1-Ra (R^2^ = 0.2121; *p* < 0.0544), and IL1-Beta (R^2^ = 0.3270; *p* < 0.0084) was found. Thus, increased visceral fat directly influences the impairment of lung function and the systemic and pulmonary immune response of women with obesity grade I.

## 1. Introduction

Obesity is recognized as a global epidemic by international health agencies, impacting over two billion individuals, or roughly 30% of the global population [1]. This growing public health challenge extends beyond obesity to include overweight, both of which are strongly linked to various health complications such as cardiovascular disease, diabetes, hypertension [2], and gastrointestinal disorders [3]. Additionally, less commonly studied comorbidities including pulmonary dysfunction and immunological impairments further underscore the complexity and breadth of obesity’s health impact.

Obesity is associated with complications and an increased risk factor for the development and impairment of lung diseases such as asthma, obstructive sleep apnea, obesity hyperventilation syndrome as well as making individuals more susceptible to respiratory inflammation, in addition to reduced lung volumes and changes in lung mechanics [4,5]. Obesity is a significant factor contributing to the reduction in forced vital capacity (FVC), a key measure of pulmonary function [6]. Excess adipose tissue, particularly in the abdominal and thoracic regions, imposes mechanical restrictions on the chest wall and diaphragm, reducing lung compliance and overall respiratory efficiency [6]. This leads to diminished lung expansion during inhalation, resulting in lower FVC values [6]. Additionally, obesity is often associated with chronic low-grade inflammation, which can exacerbate respiratory dysfunction [7]. These effects are more pronounced in individuals with severe obesity, where the combination of mechanical limitations and metabolic disturbances further compromises lung volumes, increasing the risk of conditions like obesity hypoventilation syndrome and restrictive lung disease [8].

The accumulation of fat in the abdominal region, especially visceral fat, has been associated with impaired pulmonary function, mediated by increased inflammation due to the increased number and activation of macrophages present in the visceral fat, which result in increased synthesis and the release of higher amounts of pro-inflammatory mediators, such as IL-1beta, IL-6, TNF-alpha, resistin, and leptin in the blood (serum or plasma), which can impair lung function [9].

However, considering that not only obesity, but overweight results in the increased accumulation of visceral fat, the present study investigated for the first time, whether the possible accumulation of visceral fat in overweight and not only in obesity grade I women could be associated with the impairment of lung function, lung mechanics, and pulmonary and systemic immune response. In addition, this study investigated for the first time whether this pro-inflammatory response was also present in the lungs of women with overweight and obesity.

## 2. Material and Methods

All proceedings performed in this study were approved by the ethical committee of the Federal University of Sao Paulo (registration number 3.411.606).

### 2.1. Volunteer Recruitment and Selection

Seventy-eight women were enrolled in the study and classified as follows: obesity grade I (n = 28; 45.46 ± 10.38 years old), normal weight (n = 20; 43.20 ± 10.78 years old), and overweight (n = 30; 47.27 ± 10.25 years old). All volunteers were recruited through a social program held in a public sports park in São José dos Campos, SP, Brazil, in the second semester of 2019. The socioeconomic factors were also considered, since all volunteers came from the same neighborhood (Campo dos Alemães neighborhood; with a total area of ≈ 5 square kilometers). The sample size was a convenience sample as this study was performed once as part of a social work into a very poor region of the city of Sao Jose dos Campos, SP, Brazil. The program aims to encourage physical activity, particularly among individuals with overweight or obesity grade I. The study sample consisted of women with overweight or obesity grade I who were actively engaged in the program. Participants were approached voluntarily and fully informed about the study. Afterward, they signed the informed consent form before being enrolled in the study. Although no financial incentives were provided (as it is prohibited in Brazil), the participants received free health examinations and health guidance on physical activity as part of the program. The exclusion criteria included regular practice of any type of physical activity performed at least ≥1×/week, musculoskeletal diseases, cardiorespiratory diseases, active smoking, and former smokers who quit less than 3 years prior to the beginning of the study. We specifically excluded individuals with these conditions because they are known to directly influence pulmonary function and respiratory mechanics. We acknowledge that such factors could potentially affect the outcomes, and we controlled for these factors through the initial anamnesis. Future studies may further explore the role of these additional factors in lung function in this population.

### 2.2. Anthropometric and Body Composition Analysis

Height (cm) and weight (Kg) was measured using a mechanical scale with a stadiometer (Welmy, São Paulo, Brazil). The body mass index (BMI) was calculated according to the classical formula [BMI = body weight (Kg)/heigh^2^ (cm)]. For body classification according to BMI level, we used the following: normal (BMI < 25); overweight (BMI ≥ 25); obesity I (BMI ≥ 30) [10]. Body composition was analyzed based on weight and height using octopolar multifrequency bioimpedance (Bioscan 920-II-S, Matron, UK) [11]. This bioimpedance system displays the whole body and segmented body composition (i.e., total body fat in % and in Kg, total fat free mass in % and in Kg, specific skeletal muscle in Kg, body water in % and in litters, specific skeletal muscle hydration in litter, resistance, reactance, phase angle, and impedance vector length) [11].

### 2.3. Lung Function and Mechanics

The lung function was evaluated by the spirometry test using a Master Screen spirometer (Jaeger, Frankfurt, Germany) with the forced maneuver pre and post 400 mcg of bronchodilator (salbutamol sulfate) [11,12]. The parameters evaluated were forced vital capacity (FVC), forced expiratory volume in the first second (FEV1), the relation FEV1/FVC, peak expiratory flow (PEF), and maximal expiratory flow at 25%, 50%, and 75% (MEF25%, MEF50%, and MEF75%) [11,12].

The lung mechanics were evaluated by using the impulse oscillometry system (IOS) [11,12]. This technique presents great clinical application, and is based on the evaluation of the resistance of the respiratory system at different frequencies (R5 Hz, R20 Hz, R5 Hz-R20 Hz, etc.), revealing the very specific resistance of the whole respiratory system (R5 Hz), proximal airways (R20 Hz), distal airways (R5 Hz-R20 Hz), tissue impedance (Z5 Hz), reactance (X5 Hz), and the resonant frequency (Fres) of the respiratory system. In addition, this exam identifies the resistance and the elastance of the proximal and peripheral lung tissue as independent parameters [12,13].

### 2.4. Systemic Inflammation and Immune Response

Five milliliters of venous blood was collected in a sterile vacuum tube containing anticoagulant EDTA. After the collection, 25 ul of total blood was used to perform the whole blood analysis (platelets, white and red cells) using the Sysmex XS-800i automatic blood analyzer (Sysmex, Europe GmbH, Germany). The blood tube was centrifuged at 1000× *g*, 7 min at 4 °C and the plasma was stored at −86 °C for measurements of the levels of adiponectin (DY1065), IL-10 (DY217), IGF-1 (DY291), and klotho (DY5334-05) by DuoSet ELISA (R&D Systems) using a Spectramax I3 multiplate reader (Molecular Devices, San Jose, CA, USA) [13].

### 2.5. Pulmonary Humoral Immune Response

The humoral immune response was evaluated through the measurement of adiponectin (DY1065), IL-10 (DY217), IGF-1 (DY291), and klotho (DY5334-05) in the breath condensate, which was collected using an RT Tube (Respiratory Research, Austin, TX, USA). The measurements were taken with DuoSet ELISA Kits (R&D Systems; Minneapolis, MN, USA), and the readings were carried out using a Spectramax I3 multiplate reader (Molecular Devices, San Jose, CA, USA) [13].

### 2.6. Statistical Analysis

The software GraphPad Prism 5.0 was used to perform the statistical analysis and build graphs. The one-way ANOVA followed by Tukey’s test was used to perform the comparisons among the groups. The statistical correlations were evaluated by Pearson’s test. The statistical significance was denoted for *p* < 0.05.

## 3. Results

### 3.1. Volunteers’ Anthropometric and Clinical Characteristics

Table 1 shows that there were no differences for the age and height among all groups (*p* > 0.05). On the other hand, the body weight (*p* < 0.001), body mass index (*p* < 0.001), visceral fat area (*p* < 0.001), and fat mass (*p* < 0.001) were increased when the overweight and obesity grade I groups were compared with the normal weight group. In addition, the muscle mass was increased in the overweight (*p* < 0.05) and obesity grade I (*p* < 0.001) groups when compared to the normal weight group.

### 3.2. Possible Association Between Body Composition and Lung Function and Mechanics

**Figure 1:**
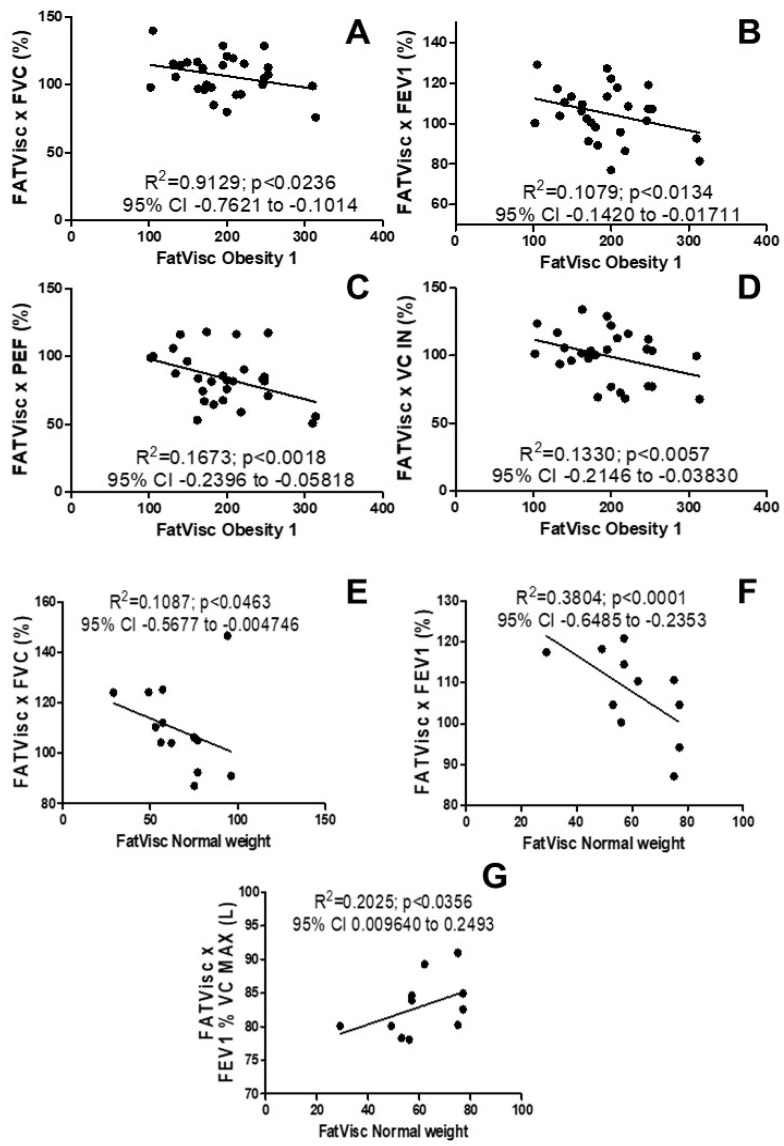
Effects of visceral fat on the lung function test (spirometry). (**A**) Correlation of visceral fat with FVC% in women with obesity grade 1; (**B**) correlation of visceral fat with FEV1% in obesity grade 1 women; (**C**) correlation of visceral fat with PEF% in women with obesity grade 1; (**D**) correlation of visceral fat with VC IN% in women with obesity grade 1; (**E**) correlation of visceral fat with FVC% in normal weight women; (**F**) correlation of visceral fat with FEV1% in normal weight women, and (**G**) correlation of visceral fat with FEV1% VC MAX (L) in normal weight women.

Figure 1 shows the possible association of body composition and pulmonary function and mechanics of women with obesity grade 1, overweight, and normal weight. It was not possible to observe a significant correlation between visceral fat and lung function in overweight women, however, the study showed a negative correlation between visceral fat and lung function in women in the obesity grade I group in Figure 1A FVC% (R^2^ = 0.9129; *p* < 0.0236; 95% CI −0.7621 to −0.1014), Figure 1B FEV1% (R^2^ = 0.1079; *p* < 0.0134; 95% CI −0.8323 to −0.2878), Figure 1C PEF% (R^2^ = 0.1673; *p* < 0.0018; 95% CI −0.6098 to 0.1887), and Figure 1D VC IN% (R^2^ = 0.1330; *p* < 0.0057; 95% CI −0.7538 to −0.08190).

A negative correlation was also presented in the group of normal weight women, as shown in Figure 1E FVC% (R^2^ = 0.1087; *p* <0.0463; 95% CI −0.5677 to −0.004746), Figure 1F FEV1% (R^2^ = 0.3804; *p* <0, 0001; 95% CI −0.6485 to −0.2353), and a positive correlation in Figure 1G in FEV1% CV MAX (L) (R^2^ = 0.2025; *p* <0.0356; 95% CI 0.009640 to 0.2493). It was not possible to observe significant correlations for the lung mechanics parameters analyzed by impulse oscillometry.

### 3.3. Possible Association Between Body Composition and Systemic Inflammation and Immune Response

**Figure 2:**
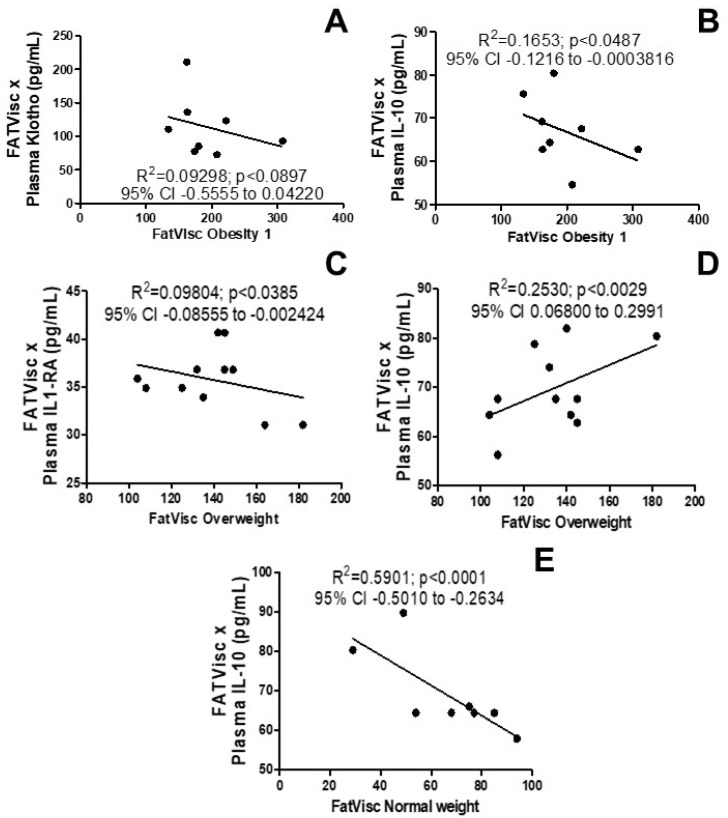
Effects of visceral fat on systemic immune response. (**A**) Correlation of visceral fat with plasma levels of anti-inflammatory and anti-fibrotic protein klotho in women in the obesity grade 1 group; (**B**) correlation of visceral fat with the plasma levels of the anti-inflammatory cytokine IL-10 in women in the obesity grade 1 group; (**C**) correlation of visceral fat with the plasma levels of the natural inhibitor of the pro-inflammatory effect of IL1β cytokine IL1-RA in overweight women; (**D**) correlation of visceral fat with the plasma levels of the anti-inflammatory cytokine IL-10 in overweight women; (**E**) correlation of visceral fat with the plasma levels of the anti-inflammatory cytokine IL-10 in the normal weight group.

Figure 2 shows the possible association between body composition and the systemic inflammations’ immune response in women in the obesity grade I group and overweight. A negative correlation among the anti-fibrotic protein klotho was observed for the first time, as shown in Figure 2A (R^2^ = 0.09298; *p* < 0.0897; 95% CI −0.5555 to 0.04220), and anti-inflammatory IL-10 (Figure 2B, R^2^ = 0.1653; *p* < 0.0487; 95% CI −0.1216 to −0.0003816) in the plasma, so in contrast to the increased visceral fat. It was also possible to observe a negative correlation in IL1-Ra, an interleukin-1 receptor antagonist that is responsible for modulating a variety of immune and inflammatory responses related to IL-1 (Figure 2C, R^2^ = 0.09804; *p* <0.0385; 95% CI −0.08555 to −0.002424), and a positive correlation in anti-inflammatory IL-10 (Figure 2D, R^2^ = 0.2530; *p* <0.0029; 95% CI 0.06800 to 0.2991) in overweight women. In addition, a negative correlation was observed in anti-inflammatory IL-10 (Figure 2E, R^2^ = 0.5901; *p* <0.0001; 95% CI −0.5010 to −0.2634) in women in the normal weight group.

### 3.4. Possible Association Between Body Composition and Pulmonary Humoral Immune Response

**Figure 3:**
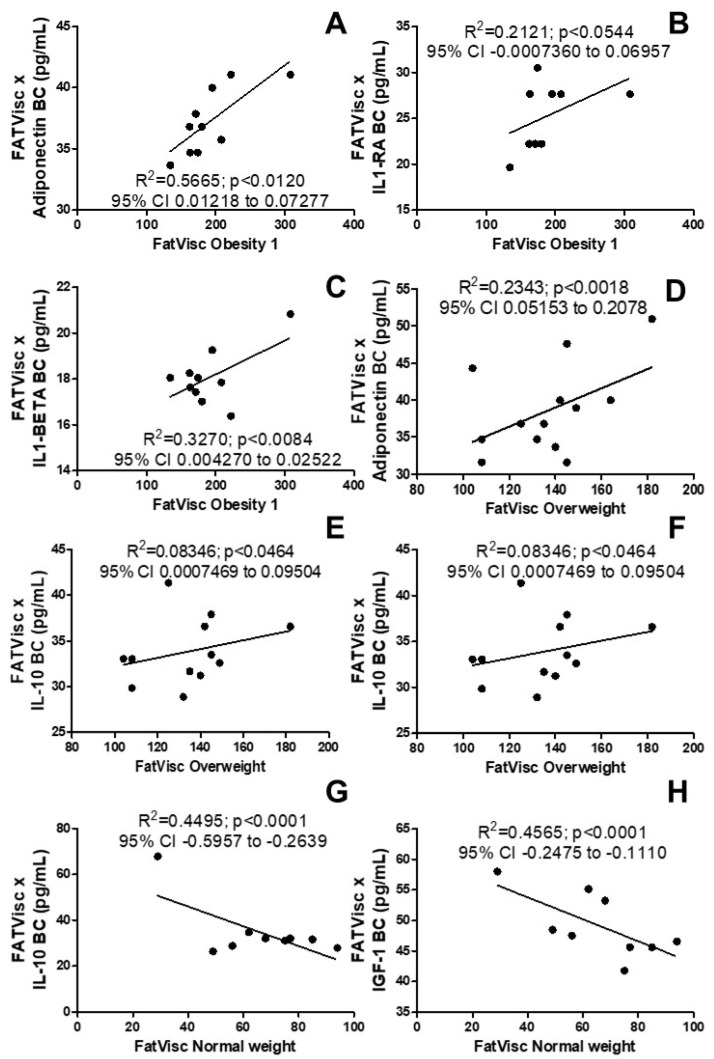
Effects of visceral fat on pulmonary immune response. (**A**) Correlation of visceral fat with the pulmonary levels of the anti-inflammatory adipokine adiponectin in women with obesity grade 1; (**B**) correlation of visceral fat with the pulmonary levels of the natural inhibitor of the pro-inflammatory effect of IL1β cytokine IL1-RA in the group with obesity; (**C**) correlation of visceral fat with the pulmonary levels of the pro-inflammatory interleukin IL1-1β in the group with obesity; (**D**) correlation of visceral fat with the pulmonary levels of the anti-inflammatory adipokine adiponectin in overweight women; (**E**) correlation of visceral fat with levels of the pulmonary anti-inflammatory cytokine IL-10 in overweight women; (**F**) correlation of visceral fat with the pulmonary levels of IGF-1 in the overweight group; (**G**) correlation of visceral fat with levels of the pulmonary anti-inflammatory cytokine IL-10 in women in the normal weight group; (**H**) correlation of visceral fat with the pulmonary levels of IGF-1 in the normal weight group.

Figure 3 shows the possible association between body composition and pulmonary humoral immune response. We observed, in contrast, the systemic inflammation immune response in the breath condensate, where there was a positive correlation for Figure 3A adiponectin (R^2^ = 0.5665; *p* < 0.0120; 95% CI 0.01218 to 0.07277), Figure 3B IL1-RA (R^2^ = 0.2121; *p* < 0.0544; 95% CI −0.0007360 to 0.06957), and Figure 3C IL1-Beta (R^2^ = 0.3270; *p* < 0.0084; 95% CI 0.004270 to 0.02522). Figure 3D also shows a positive correlation for adiponectin (R^2^ = 0.2343; *p* < 0.0018; 95% CI 0.05153 to 0.2078) and IL-10 (Figure 3E, R^2^ = 0.8346; *p* < 0.0464; 95% CI 0.0007469 to 0.09504) as well as a negative correlation for IGF-1, (Figure 3F, R^2^ = 0.1731; *p* < 0.0022; 95% CI 0.0007469 to 0.09504) in the overweight group . In addition, a negative correlation was observed in Figure 3G in IL-10 (R^2^ = 0.4495; *p* < 0.0001; 95% CI −0.5957 to −0.2639) and IFG-1 (Figure 3H, R^2^ = 0.4665; *p* < 0.0001; 95% CI −0.2475 to −0.1110) in women in the normal weight group.

## 4. Discussion

The results of the present study show a direct influence of the visceral fat on the impairment of the lung function, lung mechanics, and pulmonary and systemic immune response of grade I obese women. Such results are totally new, except for lung function, where the literature has already demonstrated a negative effect of visceral fat accumulation in a few studies [9,14,15].

Fat accumulation presents two classical characteristics: central and peripheral. Central obesity displays fat accumulation mainly in the thorax, abdomen, and visceral organs, while the peripheral displays more prominent accumulation in the hips, thighs, and subcutaneous tissue. It is well-established that visceral fat is more metabolically active than subcutaneous fat, presenting a close relation with the development of metabolic syndrome as well as an association between metabolic syndrome and respiratory diseases such as asthma and alterations in pulmonary function [4,16].

Thus, central obesity induces alterations in the respiratory pattern, characterized by increased airway and respiratory resistance, and intra-abdominal pressure [4,16,17]. Such alterations impair airflow, resulting in reduced airflow and oxygen deliverance at the alveoli level. As observed in the present study, the increases in the visceral fat were followed by reduced forced vital capacity (FVC), forced expired volume in the first second (FEV1), and peak expiratory flow (PEF) in the group of women with obesity grade I. Therefore, such impairments in FEV1 and PEF directly reflect an obstructive consequence of central obesity. In addition, reduced FVC highlights central obesity-induced restrictive disturbance. Asa a result, the present study showed that central obesity negatively affects both obstructive and restrictive respiratory patterns.

In addition, individuals with obesity present a classical pattern of chronic sub-clinical inflammation [18]. Visceral fat is the major player in the release of pro-inflammatory cytokines in the context of obesity [19]. In the present study, an increase was observed not only in the pro-inflammatory cytokines, but there was also a reduction in anti-inflammatory cytokines such as klotho and IL-10 in the women presenting central obesity. Herein, the present study showed a reduction in the plasma levels of the anti-inflammatory cytokine IL-10 with an increased rate of visceral fat in women with obesity grade I. Similarly, in the breath condensate, a reduction in IL-10 was also observed in the central obesity group as well as in the sedentary normal weight women.

Klotho is a protein with anti-fibrotic and anti-aging properties [20] and as well as IL-10 is important for controlling and reducing pro-inflammatory cytokines, and maintaining anti-inflammatory signaling [21]. Specifically, klotho possesses the capability to down-regulate IGF-1 [22]. In this way, the present study showed that obese women presented reduced plasma and pulmonary levels of klotho despite increased central obesity. Furthermore, reduced levels of klotho have been associated with lower levels of IL-10 [23], as observed in the present study. Such klotho-IGF-1-IL-10 signaling is growing as an important pathway involved in inflammatory and fibrotic diseases [24]. Beyond that, IL-1RA is an anti-inflammatory cytokine, which is the active receptor antagonist for the potent pro-inflammatory cytokine IL-1 [25]. In addition, an imbalance among IL-1 and IL-1RA has been observed in inflammatory diseases [25], and with a particularly negative correlation between the reduced plasma levels of IL-1RAd and an increase in visceral fat in overweight women [25].

Several studies have analyzed the association in the increased levels of visceral fat and reduction in plasma and serum levels of the anti-inflammatory adipokine adiponectin [26,27,28,29]. The study from Borges et. al. identified a negative correlation between visceral fat and the levels of plasma adiponectin, and beyond a positive correlation in gluteofemoral fat, which is considered as a protective fat in the context of metabolic diseases [30]. The present study went beyond demonstrating such a possible association not only systemically, but also in the lungs, as measured and analyzed in the breath condensate, particularly in women in the overweight and obesity grade I groups. These results are of note, as they revealed for the first time that the classical systemic inflammatory response in overweight and obesity is also present in the lungs, shedding important light on the role of pulmonary immune dysregulation with impaired lung function in overweight and obesity.

Furthermore, obese individuals classically present higher levels of pro-inflammatory cytokines including IL-1β, which is associated with a variety of diseases including cancer [31,32]. A positive correlation of IL-1β has been observed with the accumulation of visceral fat in women with obesity grade I. Labrecque et. al. observed an induction of pro-inflammatory IL-1β, especially in the visceral fat obtained from patients submitted to bariatric surgery [33]. In addition, IL-1β is associated with the triggering of insulin like growth factor 1 IGF-1 [8,9,10,11,12,13,14,15,16,17,18,19,20,21,22,23,24,25,26,27]. On the other hand, conflicting results are shown in the literature, some displaying higher [34,35,36], lower [37,38], or normal [39,40,41] levels of IGF-1 in overweight and obese individuals. IGF-1 is considered as an important growth factor for cardiovascular systems, while lower levels of IGF-1 may contribute to an increase in cardiovascular and cerebrovascular diseases [42]. In the present study, a negative correlation of IGF-1 was observed in the breath condensate of normal weight and overweight women.

Although the present study sheds some light on this important theme regarding how fat distribution may impact lung function and the pulmonary immune response, the present results should be considered with caution due to the small sample size, uncontrolled use of medications, dietetic assessment, and enrollment only with women, which constitutes a study limitation. An additional study limitation is related to the bioimpedance method, which is weaker in comparison to other methods such as magnetic resonance, computerized tomography, and dual energy X-ray absorptiometry. Another limitation related to the bioimpedance method, which, while practical and widely used, is that it is less accurate compared to more advanced techniques such as magnetic resonance imaging, computerized tomography, and dual-energy X-ray absorptiometry. Furthermore, there is a potential for selection bias, as the participants were recruited from a single public sports park. This recruitment strategy may have led to a sample that is not fully representative of the general population, potentially limiting the generalizability of the findings.

## 5. Conclusions

Therefore, we conclude that visceral fat, more than subcutaneous fat, may play a pivotal role in the impairment of lung function, lung mechanics, and both systemic and pulmonary immune responses in obese women. This finding underscores the distinct metabolic and inflammatory profiles of visceral fat compared to subcutaneous fat, highlighting its contribution to respiratory dysfunction. Additionally, our results reveal that the pro- and anti-inflammatory signaling typically observed systemically in individuals with overweight and obesity is also evident within the lungs. This localized inflammatory environment, particularly pronounced in obese women, suggests a potential link between adiposity, lung tissue remodeling, and immune modulation, which may exacerbate respiratory compromise in this population.

## Figures and Tables

**Table 1:** Clinical and anthropometrical characteristics.

	Normal Weight	*p* Value	Overweight	*p* Value	Obesity Grade I	*p* Value
Age	41.56 ± 10.92	*p* > 0.05	47.26 ± 10.25	*p* > 0.05	46.23 ± 10.70	*p* > 0.05
Body weight (Kg)	57.07 ± 6.47	*p* < 0.001	69.9 ± 5.29	*p* < 0.001	81.89 ± 8.85	*p* < 0.0001
Height (m)	1.57 ± 0.06	*p* > 0.05	1.57 ± 0.06	*p* > 0.05	1.60 ± 0.07	*p* > 0.05
BMI (Kg/m^2^)	22.80 ± 1.82	*p* < 0.001	28.13 ± 1.43	*p* < 0.001	32.08 ± 1.44	*p* < 0.001
Visceral fat area	81.65 ± 31.55	*p* < 0.001	140.96 ± 53.96	*p* < 0.001	199.85 ± 53.75	*p* < 0.001
Fat mass (%)	16.70 ± 70	*p* < 0.001	36.81 ± 2.87	*p* < 0.001	42.58 ± 3.56	*p* < 0.001
Muscle mass	20.99 ± 2.24	*p* < 0.05	22.78 ± 2.5	*p* < 0.05	24.27 ± 2.81	*p* < 0.001

Table 1—kilogram (Kg), meter (m), square meter (m^2^). The first *p* value corresponds to the comparison among normal weight × overweight. The second *p* value corresponds to overweight × obesity grade I. The third *p* value corresponds to normal weight × obesity grade I.

## Data Availability

The data presented in this study are available on request from the corresponding author due to institutional restriction.
